# Eugenol-Containing Essential Oils Loaded onto Chitosan/Polyvinyl Alcohol Blended Films and Their Ability to Eradicate *Staphylococcus aureus* or *Pseudomonas aeruginosa* from Infected Microenvironments

**DOI:** 10.3390/pharmaceutics13020195

**Published:** 2021-02-02

**Authors:** Joana C. Antunes, Tânia D. Tavares, Marta A. Teixeira, Marta O. Teixeira, Natália C. Homem, M. Teresa P. Amorim, Helena P. Felgueiras

**Affiliations:** Centre for Textile Science and Technology (2C2T), University of Minho, Campus de Azurém, 4800-058 Guimarães, Portugal; taniatav@2c2t.uminho.pt (T.D.T.); marta.teixeira@2c2t.uminho.pt (M.A.T.); pg35037@alunos.uminho.pt (M.O.T.); natalia.homem@2c2t.uminho.pt (N.C.H.); mtamorim@det.uminho.pt (M.T.P.A.); helena.felgueiras@2c2t.uminho.pt (H.P.F.)

**Keywords:** bactericidal, chitosan, essential oils, blended films, wound dressings, wound healing

## Abstract

Chronic wounds (CW) create numerous entryways for pathogen invasion and prosperity, further damaging host tissue and hindering its remodeling and repair. Essential oils (EOs) exert quick and efficient antimicrobial (AM) action, unlikely to induce bacterial resistance. Cinnamon leaf and clove oils (CLO and CO) display strong AM activity, namely against *Staphylococcus aureus* and *Pseudomonas aeruginosa*. Chitosan (CS) is a natural and biodegradable cationic polysaccharide, also widely known for its AM features. CS and poly (vinyl alcohol) (PVA) films were prepared (ratio 30/70 *w*/*w*; 9 wt%) by the solvent casting and phase inversion method. The film’s thermal stability and chemical composition data reinforced polymer blending and EO entrapment. Films were supplemented with 1 and 10 wt% of EO in relation to total polymeric mass. The film thickness and degree of swelling (DS) tended to increase with EO content, particularly with 10 wt % CLO (* *p* < 0.05). UV-visible absorbance scans in the 250–320 cm^−1^ region confirmed the successful uptake of CLO and CO into CS/PVA films, particularly with films loaded with 10 wt% EO that contained 5.30/5.32 times more CLO/CO than films supplemented with 1 wt% EO. AM testing revealed that CS films alone were effective against both bacteria and capable of eradicating all *P. aeruginosa* within the hour (*** *p* < 0.001). Still, loaded CS/PVA films showed significantly improved AM traits in relation to unloaded films within 2 h of contact. This study is a first proof of concept that CLO and CO can be dispersed into CS/PVA films and show bactericidal effects, particularly against *S. aureus*, this way paving the way for efficient CW therapeutics.

## 1. Introduction

Diabetes mellitus (DM) is a disabling and incurable chronic metabolic and degenerative disorder, highly prevalent in Portugal and worldwide, severely affecting patient quality of life and demanding high healthcare costs [1,2]. Diabetic foot ulcers (DFUs) are microvascular lesions that can affect the skin, soft tissues, and bones in the lower limbs [3,4]. However, more than half of those ulcers become infected, with pathogen diversity and proliferation rate within the body’s tissues determining the severity of the infection [3]. Infected DFUs are typically treated via surgical debridement [5,6,7], wound cleansing with an antiseptic solution, and antibiotic administration [8,9]. Multiple wound dressings [10,11] (e.g., films, hydrogels, foams, hydrocolloids, etc.) can then be placed over the lesion site, to protect the wound, fight infection and promote healing. Still, recurrence is frequent, with pathogen clearance and degenerated tissue recovery being increasingly more difficult each time [4]. The most commonly isolated microorganisms residing in DFUs include the Gram-positive bacterium *Staphylococcus aureus* and the Gram-negative bacterium *Pseudomonas aeruginosa*, which act as opportunistic pathogens [12,13,14]. Most of the common antibiotics, because of their excessive and inappropriate use, have induced bacterial resistance to treatment [15]. Current therapeutics are, in fact, ineffective in the treatment of most DFU-associated infections, hence the urgency for efficient alternatives. Opportunely, natural antimicrobial (AM) agents within DFU dressings have been suggested as an alternative to the set of clinically-approved conventional approaches [16,17,18,19,20,21]. 

**Figure 1:**
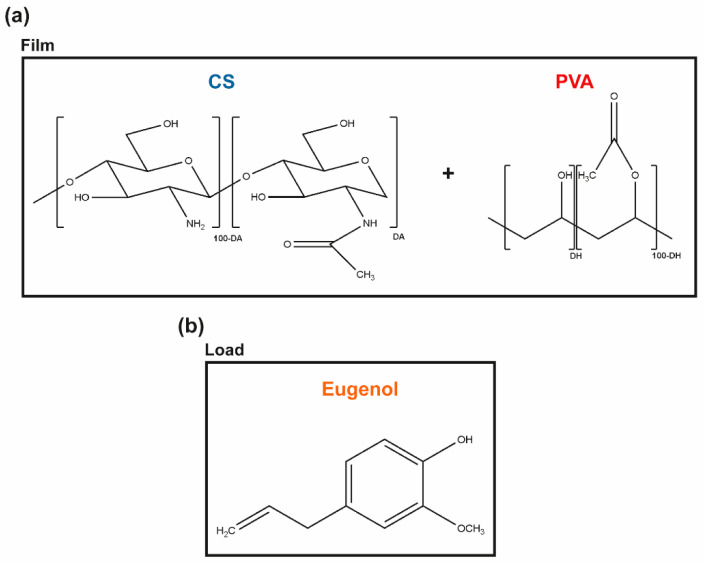
Molecular units forming the (**a**) CS and PVA polymeric matrix, and (**b**) the main phenolic component of CLO and CO, the eugenol, thereby representing the hydrophobic load.

Chitin is the second most abundant natural polymer in the world. It is the primary structural component of the exoskeleton of shrimps, crabs, lobster, and squid pens, and is present in lesser amounts in cell walls of some fungi and yeast and in plants [22]. When the degree of acetylation (DA, molar fraction of N-acetylated units) is lower than ≈ 50%, the polymer is termed chitosan (CS), carrying glucosamine and N-acetylglucosamine units connected via a β-1,4-glycosidic bond through acetal functions [23,24] (Figure 1a). CS is a nontoxic and a biologically compatible carbohydrate biopolymer, fit for multiple biomedical applications, including wound dressings. CS’s molecular weight (M_w_) and the degree of acetylation (DA) are its main structural parameters influencing the overall behavior of the polymer as a biomaterial, namely its mucoadhesive, chemoattractive, analgesic, hemostatic, and AM action (among others) [22]. Indeed, CS has a strong antibacterial activity against *S. aureus* and *P. aeruginosa*, which makes it an attractive option for the treatment of DFUs [25]. However, given its rapid biodegradation and poor mechanical properties [26], CS is frequently combined with other polymers [22,24]. 

Renewable plant-derived products with AM properties are increasingly considered as alternatives to antibiotics [20]. Essential oils (EOs), in particular, are aromatic, volatile, lipophilic biomolecules, extracted from different regions of plants, in which they work as secondary metabolites, defending the host from microbial invasion [17,20,27]. These complex mixtures contain hydrophobic molecules such as thymol, carvacrol, and eugenol (among others) that exhibit a broad spectrum of AM activity against bacteria, fungi, and viruses [20,28]. Eugenol, in particular, is an amphipathic hydroxyphenyl propene (Figure 1b), highly bactericidal towards *S. aureus* and *P. aeruginosa* [20,29]. It is also the main bioactive constituent of the cinnamon leaf (CLO ≈ 79%) and clove essential oils (CO, ≈ 81%) extracted by the Portuguese company *Folha d’Água*. CLO can be obtained through the bark and leaves of the cut trees of *Cinnamomum zeylanicum* [30], whereas CO can be obtained by distilling different parts of the plant, namely flowers, stems, and leaves of the clove tree (*Eugenia caryophyllus*) [31]. These EOs are increasingly studied for applications in the pharmaceutical and biomedical fields [19,20,28,32], due to their powerful antioxidant, anti-inflammatory, and AM properties [31,33,34], either encapsulated and/or incorporated in nanoparticles, hydrogels, films, or fibers to facilitate their delivery [19,28,35,36,37,38]. Both EOs show a promising inhibitory action on the growth of *S. aureus* and *P. aeruginosa* [19,20,39,40]. Still, their cytotoxicity at increased concentrations, their low resistance to degradation by external factors (e.g., temperature, light, moisture), and volatility in their free, liquid form hinder their expanded use [28,41]. 

In the present work, we propose to engineer films via solvent casting and phase-inversion method from CS and poly (vinyl alcohol) (PVA) blends, polymers widely combined as templates for AM action [5,42,43], and load them with the antibacterial CLO or CO to reach improved control of infections governed by *S. aureus* and *P. aeruginosa*. PVA is a biodegradable synthetic polymer produced by free radical polymerization and subsequent hydrolysis, whose chemical structure consists of a main chain formed by C-C bonds with hydroxyl and acetate groups on the sides (Figure 1). Due to its biocompatibility, biodegradability, hydrophilicity, transparency, film-forming capacity, thermal stability, and chemical resistance, PVA has been highly sought out for biomedical applications. Further, PVA’s chain flexibility strongly contributes to its biomedical versatility, despite its instability in an aqueous environment [44,45,46]. CS and PVA readily form hydrogen bonds due to a large number of -OH groups from the monomeric units of both polymers [47]. Ergo, the main goal of the present study is to explore the potential of CS and EO’s synergistic effect over microbial growth inhibition within a matrix containing the flexible and hydrophilic PVA for prospective CW treatments. Recent studies have shown that the volatile nature of EO can be protected by combining it with polymeric matrices [28]. Yet, very few have explored CS/PVA blended films as EO delivery platforms and none, to the author’s knowledge, has used the proposed approach [48,49].

## 2. Materials and Methods

### 2.1. Materials

EOs were purchased from *Folha d’Água* (Santo Tirso, Portugal) and are listed in Table 1. Trypticase soy broth (TSB), trypticase soy agar (TSA), nutrient broth (NB), and nutrient agar (NA) were acquired from VWR (Alfragide, Portugal), while Mueller Hinton broth (MHB) was obtained from CondaLab (Madrid, Spain). Bacteria were supplied from American Type Culture Collection (ATCC), encompassing Gram-positive bacteria, *S. aureus* (ATCC 6538, grown in TSB/TSA) and Gram-negative bacteria, *P. aeruginosa* (ATCC 25853, grown in NB/NA). EOs were selected based on results obtained elsewhere by the team [20,28], apart from the minimum inhibitory concentration (MIC) value of CO while incubated with *P. aeruginosa* that was here determined. In brief, the MICs of the chosen EOs–CLO and CO–against *S. aureus* and *P. aeruginosa* were determined using the broth microdilution procedure described by Wiegand et al. [50], which adapts the standard published by the Clinical and Laboratory Standards Institute (CLSI) and the European Committee on Antimicrobial Susceptibility Testing (EUCAST) [51]. EO working solutions were diluted in MHB at 40–3.7%, equivalent to 422.4–52.8 mg/mL for CO, respectively; maximum and minimum concentrations were dependent on their inherent density (Table 1). Prepared solutions were then added to the first column of 96-well plates in a volume of 100 μL. Serial dilutions (1:2) were made with MHB in the consecutive wells, to a final volume of 50 μL. Then, to each of these wells, 50 μL of the bacteria suspensions prepared at 1 × 10^7^ colony forming units (CFUs)/mL in MHB were added. EO-free bacteria suspensions and culture media were used as controls. Absorbance readings at a wavelength of 600 nm (EZ READ 2000 Microplate Reader, Biochrom, UK) were made before and after plate incubation for 24 h at 37 °C and 120 rpm.

The MIC value for each CO/bacteria combination was established as the concentration at which bacteria did not show any growth, determined visually, and confirmed by differences in absorbance readings. The existence of viable cells at the MIC and at concentrations in its vicinity (concentration higher and lower than MIC value) was determined by measuring the number of grown colonies. Briefly, aliquots of 10 µL of each cell suspension, diluted from 10^1^ to 10^5^ in phosphate buffer saline (PBS) solution, were cultured on TSA or NA plates for 24 h at 37 °C, and bacteria colonies were counted. Results are shown in Table 1. 

CS (Mw = 100–300 kDa; Acros Organics, Fair Lawn, NJ, USA) and PVA (Mw = 72 kDa, 88% hydrolyzed; Polysciences, Inc., Warrington, PA, USA) were used to produce the blended films. A DA of 9.6 ± 1.4% was determined for CS by Fourier transform infrared spectroscopy (FTIR) spectrum with KBr pellets, according to Brugnerotto et al. [52], using the N-acetylglucosamine-specific band at 1320 cm^−1^ as the measuring band, and the band at 1420 cm^−1^ as the internal reference. Aqueous solutions of glacial acetic acid (AA, Fisher Scientific, Waltham, MA, USA) and distilled water (dH_2_O) were used as solvents for CS and PVA, respectively. Sodium hydroxide (NaOH) and sodium sulfate (Na_2_SO_4_) were both acquired from Merck (Oeiras, Portugal) and included in the coagulation bath of the blends.

### 2.2. CS/EO/PVA Film Production

**Figure 2:**
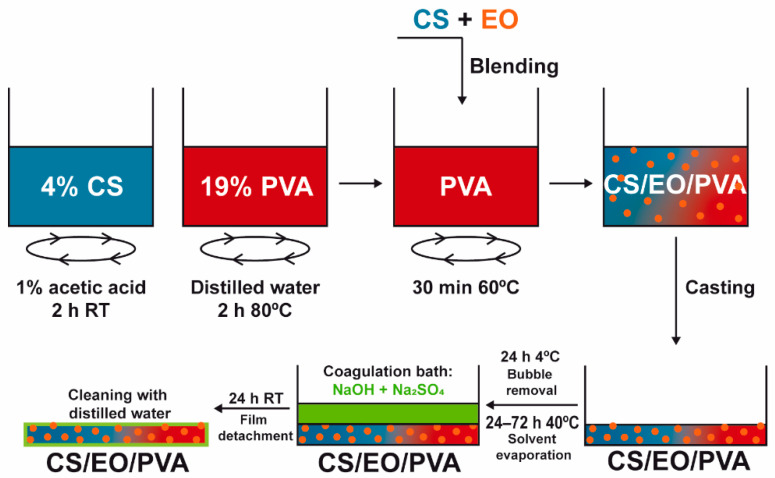
Preparation of EO-loaded CS/PVA blended films. Films were prepared through solvent casting followed by the phase inversion method, combining hydrophilic CS, and PVA to encapsulate hydrophobic EO and, this way, assist in bacterial elimination. After separate polymer dissolution in the adequate solvents, EO was added at 1/10 wt% (in relation to total polymeric mass) to the CS solution, stirred for 10 min, and blended with PVA. The casted mix was refrigerated for bubble removal, heated for solvent evaporation, and neutralized with salt ions in a suitable amount to induce film detachment from the glass. After cleaning in dH_2_O, the loaded CS/PVA/EO films were obtained.

CS and PVA films were prepared by solvent casting and the phase inversion method [17]. A 4% CS solution in 1% acetic acid was added to a 19% PVA solution (in dH_2_O at 80 °C), stirred at 200 rpm for 30 min, and casted in glass Petri dishes (Ø = 14 cm). After drying at 40 °C (24–72 h; CS-24 h; PVA-72h; CS/PVA-56 h; CS/PVA/CLO 1%–52 h; CS/PVA/CLO 10%–48 h; CS/PVA/CO 1%–54 h; CS/PVA/CO 10%–52 h) to remove excess solvent, a coagulation bath with 8% NaOH and 2% Na_2_SO_4_ was added to the samples with the goal of neutralizing and detaching the built films. After optimizing the processing methodology for each film type, a drying schedule was organized so that films could receive the coagulation bath simultaneously. Films were then kept in the later bath at 20–25 °C for a maximum of 24 h, and afterward were washed three times with dH_2_O (using an orbital shaker at 50 rpm, applied for 5 min to each wash). The latter was conducted prior to each characterization method. EO-loaded films were obtained by incorporating EO within the CS solution (already homogenous) 10 min before blending with PVA. EO was added at the necessary volume to give rise to a concentration of 1 or 10 wt% EO in regard to the total polymeric mass (specifically 35.1 or 351 mg within 3.51 g of CS and/or PVA). Figure 2 illustrates the main steps taken to produce the films, while Table 2 highlights the main built film processing conditions, along with CS/PVA mass ratios.

### 2.3. Physical and Chemical Characterization

#### 2.3.1. Macroscopic Assessment

Representative images of the films’ macroscopic structures were taken. Thickness measurements were conducted on 11 mm diameter samples of each type of film using a handheld analogical micrometer with a dial indicator from Mitotoyo (ref. 2046F, Senhora da Hora, Portugal) with a resolution of 0.01 mm, 10 mm pressing area, and 18 Pa of pressure. Film wet weight (in mg) was registered, with any excess of dH_2_O on the surface of the films being eliminated with Kimwipes (Kimtech) prior to weighting. The dried weight was collected after seven days at 37 °C, the moment at which the films’ mass reached a constant value. The films’ degree of swelling (DS, in %) was determined by measuring the weight of the samples before and after drying, similar to the process previously performed by Felgueiras et al. [17]. It was calculated using the Equation (1):

(1) $$\mathrm{DS}\left(\%\right)=\frac{m_{w}-m_{d}}{m_{w}}\times 100$$

where m_w_ (mg) is the weight of the wet film and m_d_ (mg) is the weight of the dry film. 

#### 2.3.2. Chemical Structure

The chemical structure (FTIR with attenuated total reflection, FTIR-ATR) of dried films (seven days at 37 °C) [17] was evaluated using an IRAffinity-1S, SHIMADZU spectrophotometer (Kyoto, Japan), with an ATR accessory (diamond crystal). For each sample, a total of 200 scans were performed at a spectral resolution of 2 cm^−1^, over the wavenumber range of 400–4000 cm^−1^.

#### 2.3.3. Thermal Properties

Thermal gravimetric analysis (TGA) measurements were conducted on an STA 449 F3 from NETZSCH Q500 using a platinum pan for films dried for seven days at 37 °C [17]. The TGA trace was obtained in the range of 25–700 °C under a nitrogen atmosphere, a flow rate of 200 mL/min, and a temperature rise of 10 °C/min. Results were plotted as the percentage of weight loss vs temperature. Differential scanning calorimeter (DSC) data were acquired on a Power Compensation Diamond DSC (Perkin Elmer, MA) with an Intracooler ILP, based on the standards ISO 11357-1:1997, ISO 11357-2:1999, and ISO 11357-3:1999. Tests were conducted under a nitrogen atmosphere with a flow rate of 200 mL/min and a heating rate of 10 °C/min. The thermogram was obtained in the range of 25–500 °C. Results were plotted as heat flow vs temperature.

#### 2.3.4. EO-Loaded Amount

Absorbance scans of each film were first collected between 200–800 nm (resolution of 1 nm), with a UV-2600 UV-vis spectrophotometer (Shimadzu), by resorting to an integrating sphere (ISR-2600Plus) with a film holder for transmittance analysis. Film rectangles with 6 × 3 cm^2^ were first sliced from the periphery to its center (Figure S1).

The quantity of EO loaded onto each built film was estimated via UV-visible spectroscopy using a UV-1800 UV-visible spectrophotometer (Shimadzu) in an indirect manner. Absorbance scans were equally collected, but using high precision quartz cuvettes of type 100-QS (Hellma Analyticals) and respective holder for transmittance measurements, for each one of the solutions used to detach and wash each film, right before the characterization studies. These comprised its first coagulation bath, a second coagulation bath, and three dH_2_O washing amounts, all sequentially surrounding each film during their processing. Absorbance values at 290 nm were registered, characteristic of bound eugenol within both EOs [53], and considering each solution volume, the volume occupied by each film, read absorbance values, dilution factor, and concentration from pre-determined EO-specific calibration curves, EO presence in these solutions was calculated. The estimation of detected EO mass within each film was determined after subtracting these values to the initial EO loading amount. Calibration curves relating to CLO or CO concentration in ethanol (with absorbance value ~280 nm, characteristic of eugenol within both EOs [19,54,55]) were constructed around the CLO’s MIC value of 39.3 mg/mL, previously determined against *P. aeruginosa* [20], following a 1:1000 dilution that enabled reliable detection of the spectra’s region of interest. Results were plotted as absorbance vs wavenumber.

### 2.4. Antimicrobial Action

#### 2.4.1. Agar Diffusion Assay

The Kirby-Bauer method allowed a qualitative evaluation of the antibacterial activity of the films against *S. aureus* and *P. aeruginosa* through diffusion. Briefly, bacteria inoculums were prepared in TSB and NB and left to grow overnight at 37 °C and 120 rpm. Then, their concentration was adjusted to 1.0 × 10^7^ CFUs/mL and 1 mL was collected and combined with 14 mL of TSA/NA warmed at approximately 45 °C. The 15 mL bacterial solution was poured into 90 mm diameter Petri dishes and left to solidify. Next, 6 mm diameter samples were placed on the spiked agar and were incubated at 37 °C for 24 h. Zones of inhibition (ZoI) were observed and measured to confirm the EOs’ antibacterial efficacy. Three replicate tests were carried out.

#### 2.4.2. Time–Kill Kinetics

Bacteria suspensions were prepared at 1 × 10^5^ CFUs/mL in TSB and NB and combined with all prepared films. Control groups excluded film addition. Bacteria-containing solutions were incubated at 37 °C and 120 rpm. After 0 (before action), 1, 2, 6, and 24 h of incubation, bacteria were serially diluted (10^1^ to 10^5^ in PBS), cultured on TSA/NA plates, and further incubated for another 24 h at 37 °C. Colonies of surviving bacteria were counted and reported as mean ± standard deviation (SD). Log reduction determinations were also determined between bacteria solutions with and without antimicrobial agents and unloaded and loaded films. 

### 2.5. Statistical Analysis

Statistical analysis was performed using GraphPad Prism (version: 7.04). The parametric distribution of the data was first evaluated by the D’Agostino-Pearson omnibus normality test. As the data followed a non-parametric distribution, statistical analysis was conducted with the Kruskal-Wallis test, followed by the Dunn’s multiple comparisons test, to compare each unpaired group. A confidence interval of at least 95% was chosen to define statistical significance (* *p* < 0.05, ** *p* < 0.005, *** *p* < 0.001 and **** *p* < 0.0001).

## 3. Results and Discussion

### 3.1. Macroscopic Assessment

**Figure 3:**
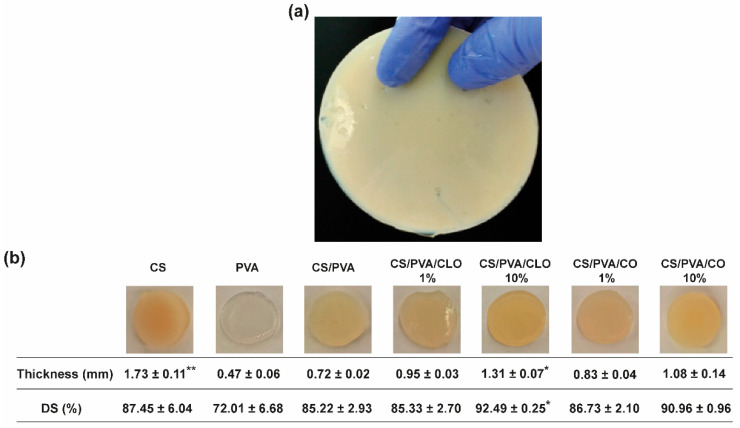
Macroscopic assessment of CS/PVA-based blended films. (**a**) Output created through the adapted solvent casting-phase inversion method, with overview hereby represented by the CS film, and (**b**) representative photographs of all built films, along with their corresponding measured thickness (mm), and gravimetrically-deduced DS (%). Results are shown as the mean ± SD (*n* = 4 punched films with 11 mm of diameter each, cut from each film’s periphery to its center). * *p* < 0.05 and ** *p* < 0.001 in comparison to the CS/PVA film, using the Kruskal-Wallis test, followed by the Dunn’s multiple comparisons test.

CS/PVA blended films were produced via the solvent casting-phase inversion method, following adaptation to what had been previously optimized by the team [17], at an optimal CS/PVA mass ratio of 30/70, reflecting an effective polymer chain entanglement, and observable macroscopic homogeneity of the films, in addition to what had already been discovered elsewhere [48]. Macroscopically smooth and homogeneous films (Figure 3) with 0.72 ± 0.02 mm of thickness and 85.22 ± 2.93% of DS were obtained, apart from occasional defects related to retained air bubbles. PVA’s highly ordered crystalline structure created the thinnest films of the group [56], having only 0.47 ± 0.06 mm of thickness, but also endowed them with high flexibility [43], as well as a soft and rubbery structure [46]. Contrarily, CS’s processing generated the thickest films, as expected from the rigid and stereoregular structure of its bulky pyranose rings that typically culminate in large free volume and less compact structures [57,58]. These films were equally inflexible and brittle, becoming even difficult to handle [49]. CS’s assembly with PVA at the 30/70 mass ratio, probably driven by hydrogen bonding, appeared to profit from both polymer’s conformational features. CS/PVA films were also more pliable [42,43], envisaging facilitated user handling and adaptation to the skin’s topography, for an improved efficacy as a wound dressing material. CLO addition tended to increase film thickness up to 131.94% or 181.94% (* *p* < 0.05), respectively, for films loaded with 1 or 10% CLO in relation to the total polymeric mass of CS and PVA. The occurrence of less condensed films suggests an alteration of the polymeric chain distribution and bonding opportunities [17]. Indeed, a substantial increment (* *p* < 0.05) of 7.27% of the overall water retention capacity of the films carrying 10% CLO was observed, suggesting a rearrangement of the polymer’s hydrophilic moieties facing outwards, providing EO-shielding within its hydrophobic core motifs [59]. CO-supplemented films predictably displayed a similar behavior, considering that both EOs are primarily composed of the same phenolic compound (i.e., eugenol), even though smaller differences were observed when compared to the unloaded control structure. Films composed of 10 wt% EO showed slightly increased pliability, though maintained adequate film integrity.

### 3.2. Chemical Structure

**Figure 4:**
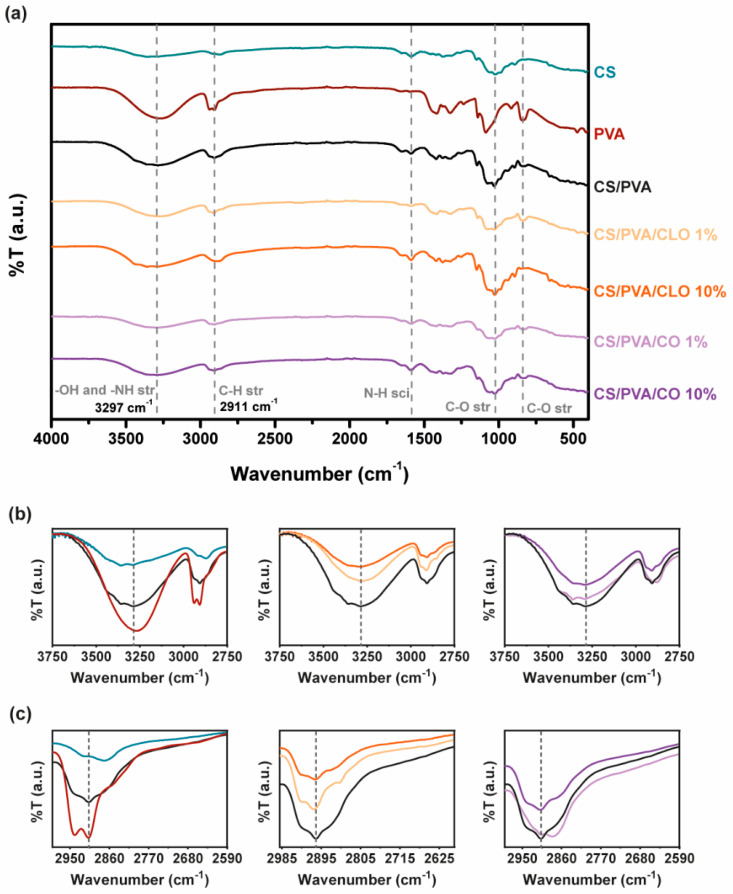
(**a**) FTIR-ATR spectra of the EO-unloaded and loaded CS/PVA-based blended films (4000–400 cm^−1^). Sections between (**b**) 3750–2750 cm^−1^ and (**c**) 2990–2590 cm^−1^ were amplified for a clearer detection of the most relevant peaks.

The characteristic saccharide peaks of CS in the 945−1190 cm^−1^ region can be observed in Figure 4, namely the C−O stretching absorption band at 1063 and 1027 cm^−1^ and the C−O−C asymmetric stretching vibrations at ∼1151 cm^−1^. The N−H scissoring deformation peak at 1594 cm^−1^, indicative of the presence of saturated primary amine groups, is also evident in the spectrum curves. Within the highlighted region, PVA absorbs distinctively at 1091 cm^−1^ (C-O stretching vibrations), additionally displaying C−C stretching vibrations at around 843 cm^−1^. CS/PVA’s spectrum shows peaks from both CS and PVA, and new peaks are absent, thereby suggesting the occurrence of polymer blending. The −NH and -OH stretching band (and intermolecular hydrogen bonding of CS backbone [60]) between 3690–2985 cm^−1^, amplified in Figure 4b, reinforces the latter assumption, suggesting that strong intermolecular hydrogen bonds of hydroxyl groups are formed between the polymers, a fact also widely identified in the literature [41,56,61]. When CS was added to PVA, the typical characteristic absorption peak of 3363 cm^−1^ shifted to a lower wave number of 3297 cm^−1^. In parallel, the polymer blends exhibited a peak with increased intensity in this region (an increase of 246%) when compared to CS alone, though shorter than the spectra of the film with 100% PVA (decrease of 38%), emphasizing fewer free -OH groups, along with an increase in interchain hydrogen bonding between the hydroxyl and amine groups of CS and the hydroxyl groups of PVA [43,49]. The broad and diffuse peak observed at 2875 cm^−1^ (CS) and asymmetric methylene CH_2_ stretching vibration at 2943 cm^−1^ (PVA) faded and slightly decreased to 2911 cm^−1^ in CS/PVA films, an effect that may be due to the C–H stretching vibrations [49].

Following EO inclusion within the films, a commitment of free –OH groups with increasing EO amount is however noticeable with both EOs, ergo suggesting that hydrogen bonds are formed between EO and the polymer chains. With CLO, the peak decreased 34% and 55%, while loaded with 1% and 10% EO, respectively, in relation to CS/PVA films. CO inclusion within CS/PVA films similarly promoted a 12% and 15% reduction in the intensity of this region of the spectra. CLO thus seem to have been better integrated in the built structures than CO, corroborating the observations made earlier from the TGA and DSC results. The section of the spectra between 3750–2750 cm^−1^ has been amplified for a clearer evaluation of these spectral variations (Figure 4b). However, in what concerns shifts of the −OH stretching vibrations with EOs, that is unclear, as also described elsewhere for similarly produced films, containing CS, PVA, and other EOs [48,49]. Among changes in C–H and C–O stretching and N–H scissoring, vibrations are also evident while comparing the spectra, as expected [49], reinforcing that bond rearrangements favoring EO incorporation took place with increasing EO loading amounts in the films, even though these are differences that are difficult to dissect on account of multiple possible contributions to the observed variations. Hints regarding a decrease in aliphatic C–H stretching vibration at 2911 cm^−^^1^ can be perceived through Figure 4c. Still, known EO-characteristic peaks, expected at 1577 and 1543 cm^−1^ (assigned to the aromatic ring C=C skeleton vibration of an aromatic substance) as well as at 1724 cm^−1^ (corresponding to the C=O stretching vibrations of the oil components [19,28]) are undetected in these spectra due to bands overlapping with the prominent 1730–1500 cm^−1^ region that contains a strong contribution of the CS’s chemical fingerprint. FTIR spectra of as-prepared hydrated films, (Figure S2), confirms results obtained.

### 3.3. Thermal Properties

**Figure 5:**
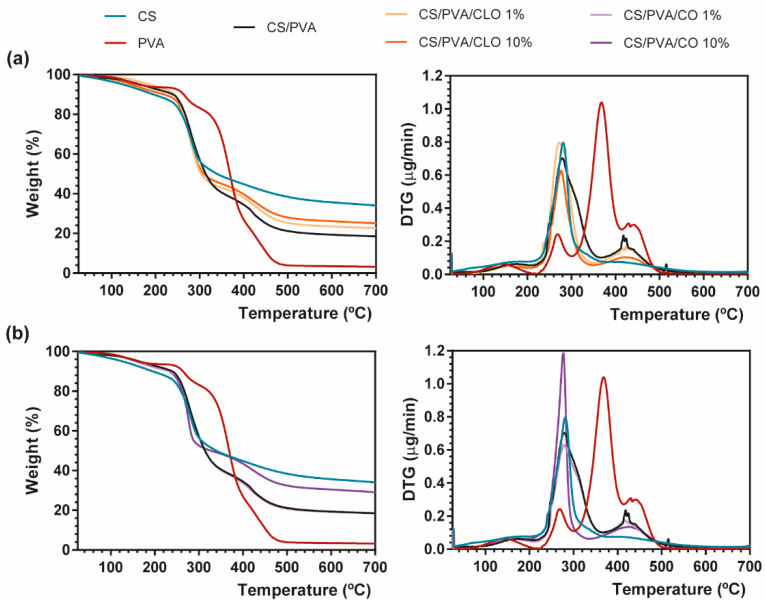
Representative TGA (left) and DTG (right) curves of (**a**) CLO- and (**b**) CO-loaded CS/PVA blended films, and respective controls from 25 to 700 °C, performed at a heating rate of 10 °C/min in a nitrogen atmosphere.

**Figure 6:**
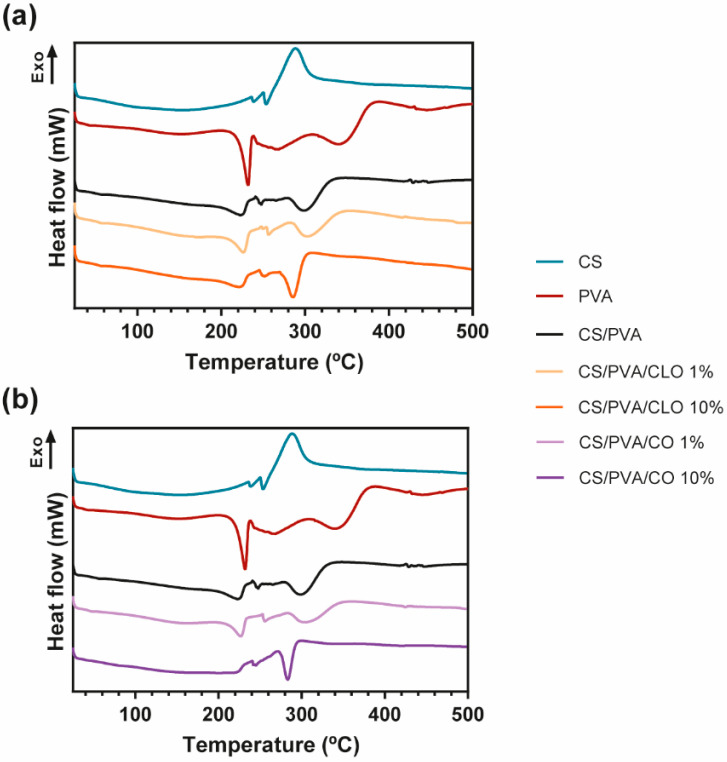
(**a**) Representative DSC thermograms emphasizing (**a**) CLO- and (**b**) CO-loaded CS/PVA blended films, and respective controls, from 25 to 700 °C, and performed at a heating rate of 10 °C/min in a nitrogen atmosphere.

Thermal properties of EO−loaded films were analyzed via TGA and DSC, specifically evaluating the effect of the EOs on thermal degradation behavior through both thermogravimetry (TG) and derivative TG (DTG) thermograms (Figure 5) and glass transition and melting phenomena (Figure 6). 

TGA measurements showed that CS has a main degradation peak at 281 °C, revealing a high residual weight (34.07%) after all the heating steps [39]. PVA film, on the other hand, exhibited four degradation steps. A first common thermal-induced modest weight loss occurred between 70–209 °C mostly due to solvent evaporation within the film, with a second degradation step detected at 220–300 °C due to PVA’s deacetylation [62]. This temperature range has also been reported as the onset of PVA’s side chain degradation. A well-defined peak arises between 300–412 °C, being assigned to the cleavage of side chains (still present), occurring the higher weight loss of PVA at 368 °C (60.16%). Finally, around 412–520 °C, the main chain of PVA polymeric backbone decomposes [63,64], until only carbon char remains (3.20% of residual mass at 700 °C). CS/PVA-related curves display the contribution of both CS and PVA’s main thermal features, corroborating the achievement of CS/PVA blended films, which occur in three distinguished steps. However, the resultant thermogram showed earlier thermal-induced degradation than the film composed only of PVA. To highlight, the loss of weight of the CS/PVA film started at around 40 °C (removal of moisture and polymer-related solvents), showing that a prominent degradation step occurs between 215 and 365 °C, with the maximum of this decomposition being clearly perceived at 280 °C (at about 50%). It corresponds to CS’s decomposition, together with the side chain decomposition and deacetylation of PVA. A second small and wider peak culminates near 412–430 °C due to the degradation of the main PVA chain [65], with only 18.48% of the sample remaining after heating up to the threshold of 700 °C. 

EO inclusion into the CS/PVA matrix, as evident from the TG thermograms, led to a decrease in weight loss compared to the unloaded CS/PVA films, indicating an enhanced thermal stability, which was probably attributed to the existence of a stronger film network favored by the interaction between polymers and EOs [66]. EO-supplemented films exhibited the same degradation profile as CS/PVA films, along with signs of an effective blending between the polymers and EOs (the presence of oils between the polymer chains and/or even linked to their chain’s groups). CS/PVA/EO films registered an earlier onset of weight loss, but simultaneously collected higher residual matter in the end, in comparison to the control. With increasing amounts of CLO in the blended structures, 23.30% and 25.08% weight remained with 1% or 10% CLO; while with matrices containing CO, 18.50% and 29.01% weight also endured after afflicted thermal variations. This phenomenon could be ascribed to the increased plasticizing effect of EOs with a rising temperature, which enhances the free volume of the chains, promotes molecular mobility, and consequently hinders the intermolecular polymer interactions and polymer–polymer interactions in the overall film network [67,68]. Films incorporated with CLO showed lower weight loss compared to those containing CO, possibly revealing stronger interactions between CS, PVA, and CLO, as also observed by Xu et al. [39]. Notwithstanding, decreasing CLO amount resulted in slightly greater weight loss compared to the 10% CLO condition, a fact that highlights the strongest interactions established between this EO and the polymers [69]. Both EOs carry a large contribution of eugenol in their composition, yet they contain a different global signature. CLO additionally contains 3.92% of β-caryophyllene and 1.91% of linalool (among other molecules present in smaller quantities), whereas CO has also 8.83% of eugenyl acetate and 8.29% of β-caryophyllene (together with numerous other trace elements); differences that certainly played an important role in the physico-chemical properties of the built films, namely its thermal-induced features. Oppositely, 10% CO loaded CS/PVA films registered a more substantial mass loss, relative to 1% CO, suggesting a weaker interaction of this EO with the polymer chains than CLO, possibly indicating as well that the addition of higher concentrations of CO increased the discontinuity of the film matrix having lower resistance to heat, with destructive consequences over the homeostasis of the ternary system [70].

The DSC curves of CS, PVA, CS/PVA, CLO-, and CO-loaded CS/PVA films are shown in Figure 6. The CS thermogram was predominantly exothermic, being an indicator of the polymer degradation process, at around 288 °C, supporting TGA results. In what concerns the PVA alone, the thermogram exhibited its glass transition temperature (Tg), near 43 °C, and a well-defined peak at 230 °C corresponding to the fusion of the most crystalline part of the polymer. This peak revealed that the crystalline structure of the polymer was maintained after film formation. In fact, all DSC curves show the permanence of the semicrystalline character of PVA, even after the production of all bioactive films. Large peaks detected at 268, 343, and 444 °C are in accordance with the results obtained from the TGA analysis, and are related to its deacetylation and decomposition of the side chains and the main chain, respectively. The control CS/PVA film thermogram exhibited a single Tg and the other thermal events (melting (Tm) and degradation temperature (Td)) were typical of both polymers, CS and PVA. While Tg of polymer blends is used in studying the miscibility and interaction between polymers, the Tm is mostly used in investigating the crystallization of polymers [71]. A single Tg of CS/PVA film appears at 56 °C, confirming the good miscibility between the polymers [72]. From the literature, to prove a good miscibility only an intermediate Tg of the applied polymers should be identified [73], even though CS’s Tg is still a subject of argument, owing to the strong intermolecular hydrogen-bonding occurring between the polysaccharide macromolecular chains [74]. The Tg of the unloaded films was higher than the one resulting from the PVA film (43 °C), uncovering a higher limitation of chain mobility because of connections established between polymeric chains [75]. The Tm relative of CS/PVA matrices was set at 225 °C, a slightly lower temperature compared to the PVA film (at 232 °C), demonstrating that the entanglement between both polymer chains alters the polymeric structure, decreasing its crystallinity (as observed in PVA film) [76]. Then, the deacetylation of PVA is noticed at 265 °C, and at 300 °C an endothermic degradation process becomes evident, being mostly due to CS’s decomposition and PVA side chains decomposition. At around 430 °C, there is a slight change in the thermogram of CS/PVA film, showing the decomposition of the main PVA chain. All these events were also seen on the TGA data, being clearly displayed in all EO-loaded film thermograms. It reveals that a homogeneous blend was also achieved in the presence of both EOs.

No changes in Tg were found in CLO-enriched films, remaining steady at 56 °C, which may indicate along with the results of TGA that CLO has a good affinity with the CS and PVA’s chains, not impairing the amorphous phase of the film. However, its Tm values and enthalpies registered a decrease as more EO was incorporated on the account of a decrease in structure crystallinity [70]. At 10% CLO loaded film, a smaller energy (25.39 J/g) was required to degrade the matrix crystalline regions, comparatively to 1% CLO loaded film (35.59 J/g) and unloaded films (34.59 J/g). These results undoubtedly emphasize that the presence of EO between the chains, particularly with larger incorporated quantities, left some free space between the chains. The peak relative to the degradation in the highest EO concentration also required greater energy for the degradation to take place, confirming that EO incorporation was efficient, even at 10% CLO. The different events within the CO’s film thermogram also demonstrated efficient EO incorporation within the CS/PVA matrix. The Tg from 1% CO suffered a slight reduction, accentuating the presence of CO between the amorphous polymeric phase, thereby enabling easier chain mobility. The Tm and enthalpy values associated increased (227 °C and 40.74 J/g, respectively) comparatively to control CS/PVA film, due to the oil presence between the polymeric chains, but not connecting to them as effectively as CLO. Regardless, 10% CO loaded film thermogram corroborates TGA findings, since there was no detection of Tg. Also, a prominent difference was registered in the temperature range corresponding to the polymeric Tm, which was detected at 245 °C, but within a much less evident peak and requiring much lower energy to occur (13.34 J/g). These camouflaged events denote that this EO does not have as good an affinity for the polymeric chains as CLO, being present in the film in a more disorganized way.

### 3.4. EO-Loaded Amount

**Figure 7:**
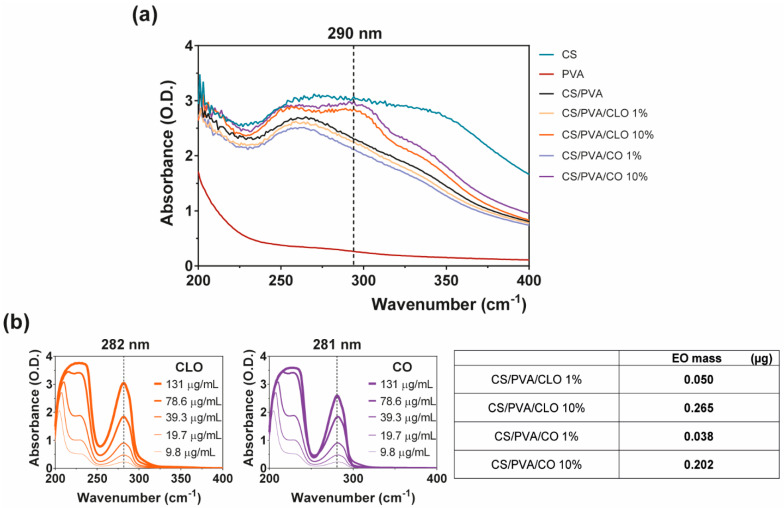
EO loaded amount within CS/PVA-based blended films. (**a**) UV-visible spectroscopy curves of CS/PVA-based blended films, highlighting wavelength of 290 nm showcasing entrapped or bound EO [53]; (**b**) characteristic curves of free CLO and CO at multiple concentration values, highlighting the wavelength of 280 nm as EO representative [19,54,55] (left), along with a table (right) comprising representative data on EO mass (µg) within the loaded film samples of 6 mm in diameter, obtained via an indirect route by analyzing EO presence within film coagulation baths and dH_2_O washing volumes, all sequentially surrounding each film during their processing. Results included data from representative films.

The successful loading of CLO and CO into CS/PVA films during film processing was confirmed by UV-visible spectroscopy, with the absorption spectra being recorded over wavelengths ranging from 200 to 800 nm, thereby including all relevant phenomena of light absorption/scattering from the samples. The wavenumbers 282 and 281 nm, respectively for CLO and CO (Figure 7b, left), constituted the most distinguishable wavenumbers carrying a peak of light absorbance characteristic of the EOs. Coincidently, eugenol absorbs at 280 nm due to its condensed benzene ring system [19,54,55]. Since eugenol is the main contributor to both CLO and CO’s composition, this was taken as a reference for results interpretation. Absorbance scans of increasing EO solution concentrations allowed to verify that (Figure 7b, left), additionally enabling the determination of EO-specific calibration curves that were then used to calculate EO concentration within the films.

EO-loaded films at 10% displayed a maximum absorption peak at ~290 nm, as expected (Figure 7a). CS’s monomers are known to absorb light in this region of the spectra [77]. PVA films, on the other hand, are transparent in the UV–visible region, which results in a very low absorption level [78], with both CS and EO addition contributing to an increase in film opacity and light scattering phenomena [53]. The shift from the ~280 nm absorption peak of the free oils to the ~290 nm of loaded films is, therefore, another indicator of the strong binding denoted between film components and the entrapment/distribution of the oil molecules within the polymeric matrix. Visualization of EO contribution within 1% loaded films was not possible. As observed from Figure 7b (left), as the concentration of the oil decreases below 10 µg/mL, recognition of the peak becomes more challenging. Thus, considering that via the indirect route only 0.050 µg/mL of CLO and 0.038 µg/mL of CO were found (Figure 7b, right), this would be expected due to equipment sensitivity. Still, data from FTIR and TGA/DSC analyses recognized the existence of the EOs in the films. The loading amount of CLO and CO is very similar, though CLO appeared to have incorporated slightly more EO. This strengthens the observations made previously in which it was stated the attraction of the CLO towards the polymers and the more resilient interactions generated. Here, we confirm that the improved interactions between CLO and CS/PVA are not an effect of its increased presence in the film but rather an enhanced affinity. It would be expected that the loading amount achieved from 10% EOs solutions to be 10 times superior to that attained with 1% solutions. Yet, that is not the case. Data reports an early saturation of the films with 0.9% of the initial loading solution. Binding via the hydroxyl groups of the polymer was promoted, facilitating the retention of EO-related molecules. However, due to the porous nature of the films, which increases water infiltration, and the polymers’ large affinity towards these molecules (Figure 3), it is also likely that competition for these groups takes place, with many –OH radicals being blocked by water [17,57]. 

### 3.5. Antimicrobial Action

#### 3.5.1. Agar Diffusion Assay

**Figure 8:**
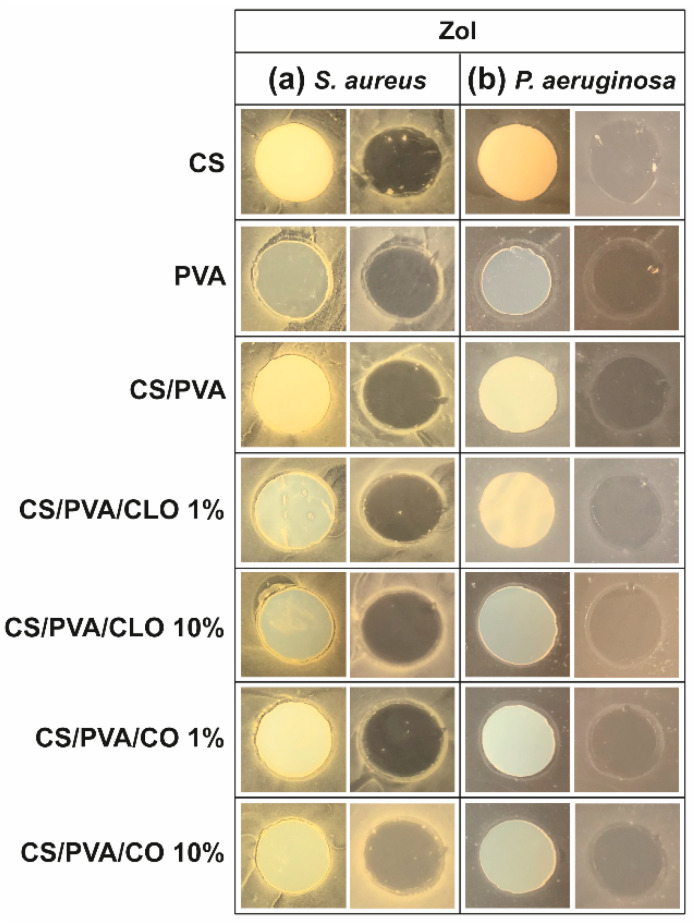
ZoI of unloaded and EO-loaded CS/PVA blended films while cultured with (**a**) *S. aureus* and (**b**) *P. aeruginosa* bacteria, up to 24 h. Images were collected without regard for size proportionality, being only used to reveal the halos formed. For each bacterium, left images depict films at their original location at the beginning of the assay, along with the bacteria that grew over the incubation period; while on the right, cultured films were carefully removed from the agar so that contact-kill could be visualized.

The antibacterial activity of unloaded and EO-loaded CS/PVA films was assessed against the Gram-positive bacteria *S. aureus* and the Gram-negative bacteria *P. aeruginosa* via the agar diffusion test. Data from Figure 8 shows the observed ZoI in the presence of the film, before and after its removal from the agar plate. ZoI measurement was not performed due to its feeble presence; however, this is not a statement of low AM activity. As such, data from Figure 8 can only be considered as indicative of the AM potential of the CLO and CO-loaded CS/PVA films. AM activity is materialized via multiple modes of action, either acting collectively and independently [79,80]. CS film is AM by direct contact with the bacteria, a fact that is evident by observing the corresponding images on the right side of both (a) and (b) sections, particularly with *S. aureus*. Weak image contrast in *P. aeruginosa* data hinders bacteria visualization. Solid CS can display AM activity over a broader pH range than its soluble and diffusible form. As pH is above pKa (typically near 6.5, though tunable with DA and Mw variations [57]) in standard bacterial cultures such as these, the inhibitory effect is exerted by hydrophobic interactions and chelating capacity of divalent metal ions in neutral conditions rather than electrostatic interactions between its protonated amines and anionic bacterial outer layer structures [80]. PVA film’s antibacterial action, surprisingly, was revealed in the *P. aeruginosa* microenvironment by a thick bacteriostatic ring outside the film’s periphery, even though PVA is not traditionally linked to an antibacterial effect. Regardless, the antibacterial action of the unloaded blended films, containing 30% CS and 70% PVA, remained unattractive. 

With EO-loaded CS/PVA films, a higher bioactivity was expected, since multiple antimicrobial compounds co-exist within EO’s structure, namely low molecular weight phenols, terpenes, and aldoketones [28]. In fact, a discrete improvement of their efficacy was detected, particularly with *S. aureus*, exhibiting a thin but visible clean bactericidal ring followed by an also thin but intense spiked ring (bi-phasic action) that typically contain interaction precipitates and repelled bacteria [81]. In the Gram-negative specie, a thicker bacteriostatic ring became exposed, ergo, some activity against these microorganisms is also observed through this characterization method. The superior activity against *S. aureus* than *P. aeruginosa* can be explained by the differences between Gram-positive and Gram-negative bacteria at the level of the architecture and molecular components of their cell wall. In the case of Gram-negative, two distinct lipid membranes, the cytoplasmic and the outer membrane, exist, thus forming a forceful inward/outward barrier. The absence of the latter in the Gram-positive peripheral structure, containing only a bilayer membrane (cytoplasmic), allows a higher facility for an antibacterial agent’s perfusion through its cellular wall [20,82]. Still, no clear differences can be perceived between CLO and CO, neither with higher nor lower EO amounts. The EOs are mainly within the films and not on their surface, as such it is conceivable their hydrophobic nature to cause a poor EO diffusion through the film network under static conditions. Further, the agar tortuosity may also hinder the EO diffusion, which can be as well influenced by the oil density (Table 1). CO is slightly denser than CLO; yet, in both cases, ZoI were very difficult to identify. Even if tenuous, 10% CLO and 1% CO addition to the blends appears to have induced a stronger AM effect. Eugenol is an amphipathic hydroxyphenyl propene highly bactericidal towards *S. aureus* and *P. aeruginosa* [20,29], known to interfere with the cells intracellular functions or ions transport, preventing important metabolites and connection pathways from taking place and, ultimately, leading to the cell death [19]. However, the aforementioned differences in CLO and CO’s composition most certainly played as well an important role in the biological properties of the built films, despite all of these molecules being considered AM [79,82,83,84,85].

#### 3.5.2. Time–Kill Analysis

**Figure 9:**
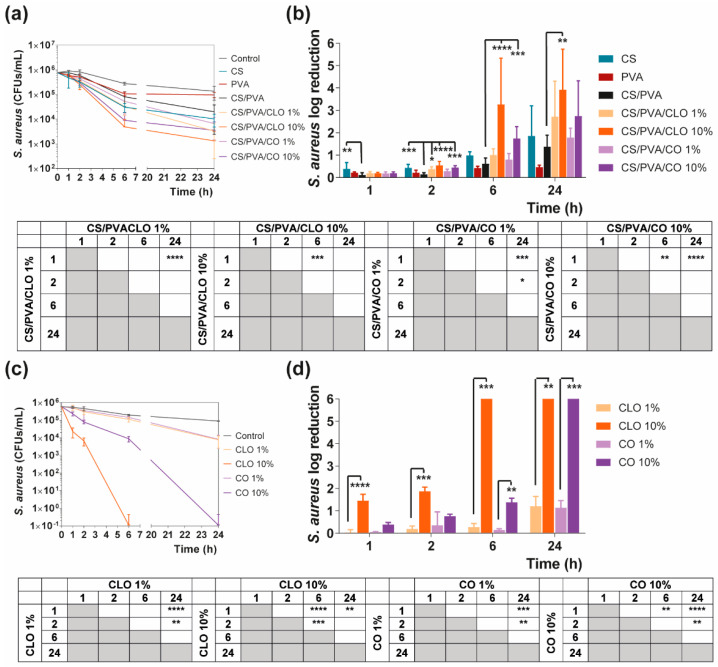
Time–kill curves of (**a**) unloaded and EO-loaded films and (**c**) EOs at film loaded concentration, against *S. aureus* bacteria, up to 24 h of culture. Positive controls for *S. aureus* (growth without agent or film) were also conducted (grey line), reaching a maximum value of ≈ 8.9 × 10^6^ CFUs/mL after a 24-h culture (data not shown in graphic). *S. aureus* reduction (calculated as log reduction) of (**b**) the films and (**d**) EOs in relation to control samples. The elimination of 100% of bacteria was considered as log 6. Results are represented as the mean ± SD (*n* = 3). Statistically significant differences can be highlighted, * *p* < 0.05, ** *p* < 0.005, *** *p* < 0.001, and **** *p* < 0.0001, for each sample at each time point in comparison to (**c**) each free EO or to the (**d**) loaded CS/PVA films, using the Kruskal-Wallis test, followed by the Dunn’s multiple comparisons test. The evolution over time of each EO and EO-loaded film’s AM action was also examined and shown in a table format for clarity.

**Figure 10:**
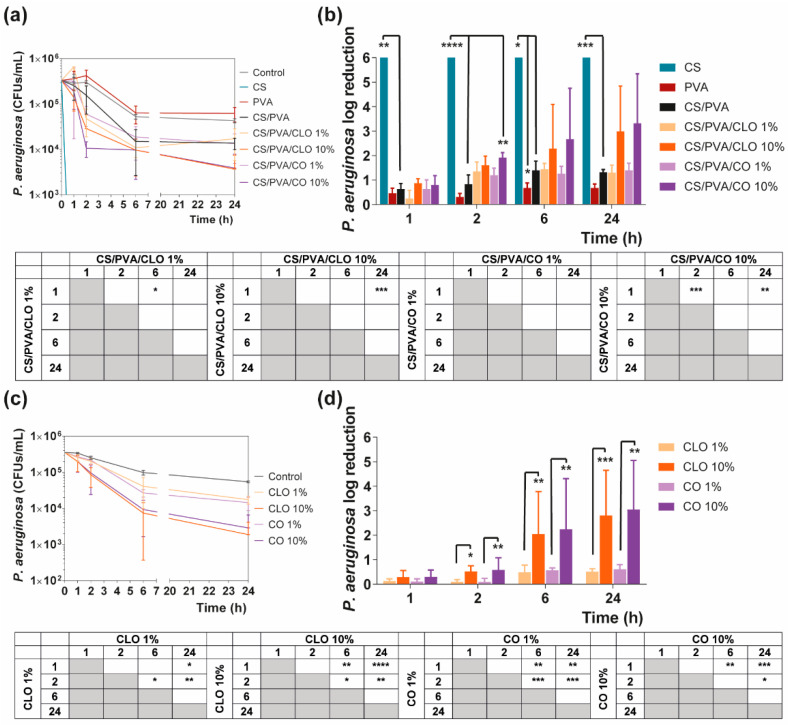
Time–kill curves of (**a**) unloaded and EO-loaded films and (**c**) EOs at film loaded concentration, against *P. aeruginosa* bacteria, up to 24 h of culture. Positive controls for *P. aeruginosa* (growth without agent or film) were also conducted (grey line), reaching a maximum value of ≈ 4.3 × 10^6^ CFUs/mL, after a 24 h culture (data not shown in graphic). *P. aeruginosa* reduction (calculated as log reduction) of (**b**) the films and (**d**) EOs in relation to control samples. The elimination of 100% of bacteria was considered as log 6. Results are represented as the mean ± SD (*n* = 3). Statistically significant differences can be highlighted, * *p* < 0.05, ** *p* < 0.005, *** *p* < 0.001, and **** *p* < 0.0001, for each sample at each time point in comparison to (**c**) each free EO or to the (**d**) loaded CS/PVA films, using the Kruskal-Wallis test, followed by the Dunn’s multiple comparisons test. The evolution over time of each EO and EO-loaded film’s AM action was also examined and shown in a table format for clarity.

Quantitative data regarding growth inhibition of *S. aureus* and *P. aeruginosa* while incubated with each one of the unloaded and EO-loaded films under dynamic conditions was evaluated by the time–kill kinetics. Here, the number of remaining viable colonies at specific incubation periods (1, 2, 6, and 24 h) and the respective log reduction was determined for unloaded and EO-loaded CS/PVA films (Figure 9 and Figure 10). Films created with 30% CS, 70% PVA, and 1/10% CLO/CO were capable of invoking a higher AM activity than the control without any agents, as expected. The suspicions around EO’s benefits, evoked by the agar diffusion assay, have been amplified with this test. Its inherent dynamic conditions may have promoted EO’s mobility throughout the polymeric network and facilitated bacterial encounters for a more efficient antimicrobial action. Additionally, for both microorganisms, the bactericidal action of 100% CS films was observed from the first hour of contact (** *p* < 0.005), being particularly devastating for *P. aeruginosa* with total inhibition right from the first chosen time point of 1 h. Despite the complexity of the Gram-negative bacteria cell wall, CS was extremely effective. Similar observations were made by Tin et al. when testing different CS and CS-derivatives against commercial and clinical strains of *P. aeruginosa* [86]. Predictably, after blending with PVA, the overall activity decreased significantly. CS/PVA films comprise the same total mass, but only 30% of CS. Thus, many of the cationic groups of CS, which are responsible for the strong interaction with the electronegative surface of the bacteria and, consequently, its AM activity, became entrapped within the matrix being less available for cell binding. 

In the case of EOs, their action begins after 2 h of contact with each of the bacterium. In *S. aureus*-enriched cultures, films impregnated with CLO at 10 wt% were the most effective from the group. CS/PVA/CLO 10% induced a greater log reduction than CS/PVA matrices, following a 2 h (**** *p* < 0.0001), 6 h (**** *p* < 0.0001) and 24 h (** *p* < 0.005) incubation. Its activity over time (Figure 8b) clearly intensified at 6 h (*** *p* < 0.001) in relation to the first time point. The CLO at 1 wt% also exhibited a promising antibacterial action at 2 h (* *p* < 0.05) though blurred with increased incubation time. CO-supplemented films showed a similar profile, though slightly weakened and inconsistent. Still, statistically significant differences were determined at the time points of 2 h (*** *p* < 0.001) and 6 h (*** *p* < 0.001). With these films, an intensified action was evident at 6 h (** *p* < 0.005) but even more following the 24 h (**** *p* < 0.0001). Importantly, equivalent experiments with only EOs as bioactive agents (Figure 8b) and added to the cultures, with EO concentration as estimated through the results depicted in Figure 7b (concentration of EO within post-processed films), followed the same trend as aforementioned for the films, confirming the results obtained. The main observed difference was that 10 wt% CLO started its activity within 1 h (**** *p* < 0.0001 in respect with 1 wt% CLO) of contact with the established bacterial settings, with 1 wt% EO causing a null log reduction effect. On the other hand, while facing *P. aeruginosa*, EO-loaded films also tend to reduce the number of viable bacteria more than films devoid of these biomolecules, with CO appearing to have increased AM potential. Nevertheless, no statistically significant differences were seen apart from increased antibacterial activity of CS/PVA/CO 10% at the time point of 2 h. All the same, cultures with the EOs alone corroborated, again, results obtained with EO-containing films.

## 4. Conclusions

Films made up of CS and PVA incorporated with CLO and CO are here proposed for the treatment of DFUs, a microenvironment rich in *S. aureus* and *P. aeruginosa* bacteria. The incorporation of these two EOs in the CS/PVA film has the potential to increase the AM activity conferred by the CS, since the combination with CLO and CO increased the films’ overall antibacterial effects against the two tested strains. 

CS and PVA have a good capacity to form intermolecular hydrogen bonds due to a large number of inherent hydroxyl groups within their structures. CS/PVA blended films were successfully produced, with CS bestowing AM properties to the construct, and PVA adding flexibility and hydrophilic capacities. Even though both EOs were successfully encircled by the polymeric matrix, CLO incorporated 31% more EO than CO, an amount that appeared to be also bound to the polymer chain in a more effective way. In 1 h, films built solely of CS showed antibacterial signs against *S. aureus* and were totally effective in eliminating *P. aeruginosa* from the culture vessels. However, 100% CS films lacked mechanical properties enabling an adequate handling and applicability. CS/PVA films supplemented with CLO or CO were mechanically fit, showing efficient bactericidal effects following 2 h of direct contact within the infected microenvironments, being significantly more efficient than unloaded films. 

Future work will be directed towards a balance between AM action of CS and its mechanical hindrance after processing, together with the combination with CLO or CO for an intensified antimicrobial profile against both bacteria.

## Figures and Tables

**Table 1:** List of tested EOs, their origin, density, and MIC values in relation to tested *S. aureus* and *P. aeruginosa* reference strains.

EO	Abbreviation	Origin	Density (g/cm^3^)	MIC (mg/mL)	
				*S. aureus*	*P. aeruginosa*
Cinnamon leaf	CLO	*Cinnamomum zeylanicum*	1.049	0.82	39.3
Clove	CO	*Eugenia caryophyllus*	1.056	0.83	52.8

**Table 2:** Data required to build tested CS/EO/PVA blended films, specifically EO loading amount (in µL), mass (g), and volume (mL) of polymer solutions for each case, total mass percent (%*w/v*), total volume (mL), and selected CS/PVA mass ratios.

	EO		CS Solution		PVA Solution		Total % *w*/*v*	V_Total_ (mL)	CS/PVA Mass Ratios
	m (mg)	V (µL)	m_CS_ (g)	V (mL)	m_PVA_ (g)	V (mL)			
CS	-	-	3.51	39	-	-	9	39	100/0
PVA	-	-	-	-	3.51	39	9	39	0/100
CS/PVA	-	-	1.05	26	2.46	13	9	39	30/70
CS/PVA/CLO 1%	35.1	39.2	1.05	26	2.46	13	9	39	30/70
CS/PVA/CLO 10%	351.0	392.0	1.05	26	2.46	13	9	39	30/70
CS/PVA/CO 1%	35.1	33.2	1.05	26	2.46	13	9	39	30/70
CS/PVA/CO 10%	351.0	332.0	1.05	26	2.46	13	9	39	30/70

4% CS and 19% PVA solutions were used.

## Data Availability

Not available.

## Supplementary Materials

The following are available online at https://www.mdpi.com/1999-4923/13/2/195/s1, Figure S1: Macroscopic view of CS/PVA-based blended films. Representative photographs of sliced rectangles (from the periphery to its centre) with 6 × 3 cm^2^, used for UV-visible spectroscopy measurements, Figure S2: FTIR-ATR spectra of the EO-unloaded and loaded CS/PVA-based blended films (4000-400 cm−1), in a (a) hydrated (as-prepared), and (b) dried state (after drying for 7 days at 37 °C).
