# Supplementary material for: Eugenol-Containing Essential Oils Loaded onto Chitosan/Polyvinyl Alcohol Blended Films and Their Ability to Eradicate Staphylococcus aureus or Pseudomonas aeruginosa from Infected Microenvironments
Source: Pharmaceutics. 2021 Feb 2;13(2):195. doi: 10.3390/pharmaceutics13020195 (PMC7912801; doi:10.3390/pharmaceutics13020195)
Supplement: Supplementary file 1 [file pharmaceutics-13-00195-s001.pdf]

pharmaceutics

Supplementary

Eugenol-containing essential oils loaded onto chi-tosan/polyvinyl alcohol blended films and their ability to eradicate Staphylococcus aureus or Pseudomonas aeruginosa from infected microenvironments

Joana C. Antunes, Tânia D. Tavares, Marta A. Teixeira, Marta O. Teixeira, Natália C. Homem, M. Teresa P. Amorim and Helena P. Felgueiras

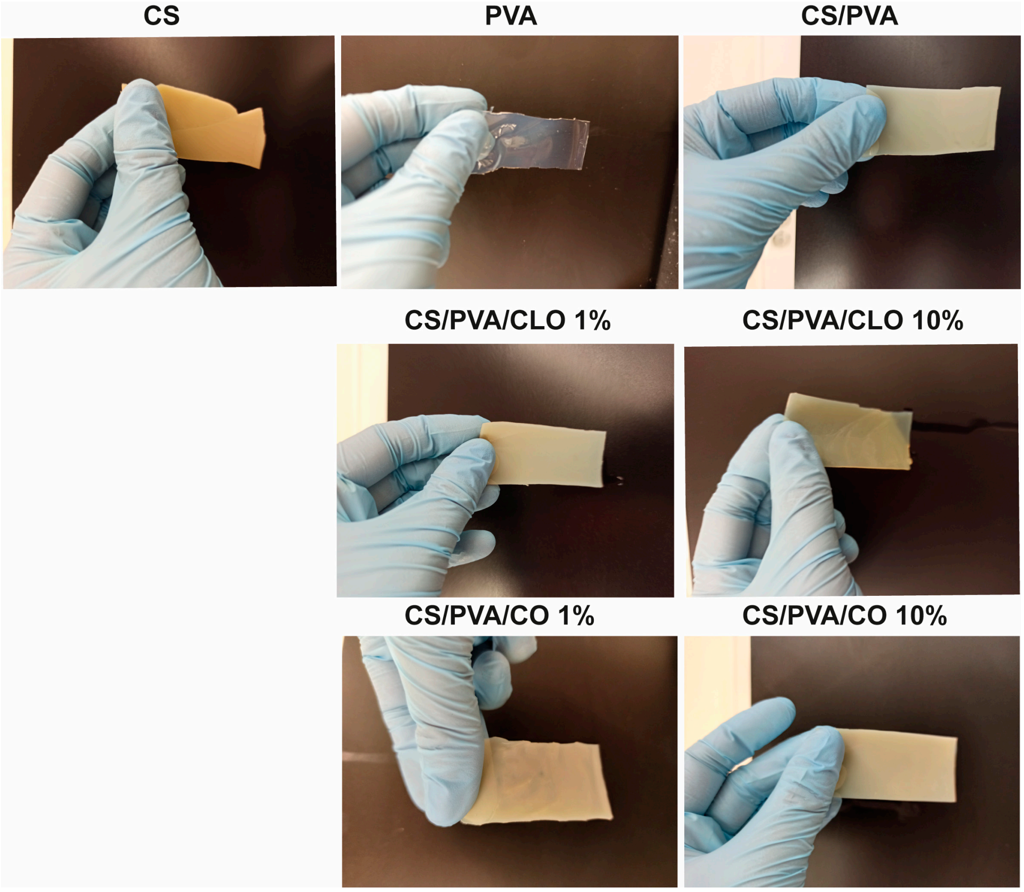

Figure S1. Macroscopic view of CS/PVA-based blended films. Representative photographs of sliced rectangles (from the periphery to its centre) with 6 × 3 cm², used for UV-visible spectroscopy measurements.

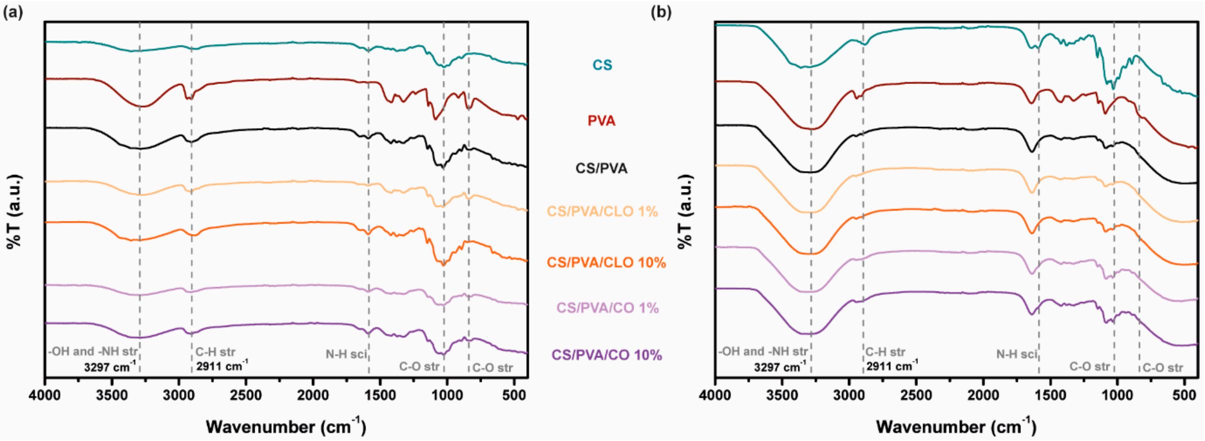

Figure S2. FTIR-ATR spectra of the EO-unloaded and loaded CS/PVA-based blended films (4000-400 cm^{-1}), in a (**a**) hydrated (as-prepared), and (**b**) dried state (after drying for 7 days at 37 °C).
